# Eye Movements in Medical Image Perception: A Selective Review of Past, Present and Future

**DOI:** 10.3390/vision3020032

**Published:** 2019-06-20

**Authors:** Chia-Chien Wu, Jeremy M. Wolfe

**Affiliations:** 1 Visual Attention Lab, Department of Surgery, Brigham & Women’s Hospital, 65 Landsdowne St, Cambridge, MA 02139, USA; 2 Department of Radiology, Harvard Medical School, Boston, MA 02115, USA; 3 Department of Ophthalmology, Harvard Medical School, Boston, MA 02115, USA

**Keywords:** visual search, eye movements, medical image perception

## Abstract

The eye movements of experts, reading medical images, have been studied for many years. Unlike topics such as face perception, medical image perception research needs to cope with substantial, qualitative changes in the stimuli under study due to dramatic advances in medical imaging technology. For example, little is known about how radiologists search through 3D volumes of image data because they simply did not exist when earlier eye tracking studies were performed. Moreover, improvements in the affordability and portability of modern eye trackers make other, new studies practical. Here, we review some uses of eye movements in the study of medical image perception with an emphasis on newer work. We ask how basic research on scene perception relates to studies of medical ‘scenes’ and we discuss how tracking experts’ eyes may provide useful insights for medical education and screening efficiency.

## 1. Introduction

Detection and diagnosis in medicine are frequently based on analysis of medical images. Clinicians of various specializations consume a vast volume of medical images each day. They perform remarkable tasks with these images but they are not perfect. For instance, though more than two million new cases of breast cancer and lung cancer were diagnosed worldwide in 2018 according to the report from World Cancer Research Fund (worldwide cancer data, https://www.wcrf.org/dietandcancer/cancer-trends/worldwide-cancer-data), we know that many cancers are not discovered, even though they may be visible in the image (e.g., [1,2,3,4]). Though the acceptance of routine cancer screening has risen and the imaging technology has continued to advance, false negative and false positive rates continue to be higher than we would expect or desire [5,6]. Measuring the eye movements of experts as they perform medical image perception tasks is one way to identify possible weak spots in the processes of medical image perception, raising the possibility of interventions that could improve performance. Eye tracking can also be used to assess the effectiveness of those interventions. Finally, from the vantage point of the basic science of perception, expert performance with medical images can give us insight into the processes of more ordinary acts of scene perception.

One of the interesting aspects of medical image perception research is that the stimuli keep changing. Fifty years ago, questions about medical image perception would have been questions about static 2D, achromatic x-ray images, presented on film. Today, technologies, like CT, create a set of virtual slices through some volume of the body (e.g., the chest) and produce a 3D volume of image data to be examined [7]. They could look at nuclear medicine images (e.g., positron emission tomography—PET) or ultrasound [8], where a 3D dataset might be rendered as a rotating figure. Furthermore, many images are in color today [9]. The dataset might be 4-dimensional, with a time-varying fourth dimension as in CT angiography where a contrast agent is injected into the bloodstream and tracked in 3D as it sweeps the heart, brain, etc. [10]. Thus, there are three spatial dimensions and the images evolve over time [11], creating 4D datasets.

Each advance in technology requires (or should require) a new set of psychophysical studies because each new modality presents new opportunities and challenges for the perceptual and cognitive capabilities of the observers. What we learned about search strategies or patterns of errors in 2D films may be only loosely applicable to newer forms of imaging. It is nearly impossible to develop better viewing strategies without understanding how human perception works in these new modalities.

Though the technology changes, many of the basic perceptual issues do not. Kundel, Nodine and their colleagues and students have worked for many years on a set of issues that remain relevant today. We will organize this brief review starting with the scanpaths that can be measured during visual search, since the sequences of eye movements are the basic data that is collected in eye movement studies in medical image research. Once scanpaths are collected, they can be aggregated in various ways to address other questions such as the extent of the “useful field of view” (e.g., How much of the image can be processed around the current point of fixation?) and the nature of search errors (e.g., Was the missed cancer fixated during search?). The topic of “satisfaction of search” is an extension of the topic of search errors. Finally, we will discuss what people can perceive in a single glimpse—the ‘gist’ that can be extracted when there is no scanpath at all.

For other important topics in the field of eye movements in medical image perception (e.g., medical training and education), there are other useful reviews: For example, [12,13,14].

## 2. Scanpaths: Searching in Scenes and Medical Images

The sequence of saccades and fixations made when an observer views an image is known as the “scanpath” [15,16]. It has long been used as a clue to what observers are doing when they are looking at something. Essentially all eye movement studies begin by recording a set of scanpaths. Scanpaths tend to be substantially different each time an image is viewed. Thus, for many of the studies described below, scanpaths are aggregated in ways that lose the precision of space and time that a single scanpath would possess. For example, it is often useful to measure the likelihood that a specific location in the image was fixated in the scan paths of many observers. In such an analysis, temporal order will be sacrificed to create a spatial map of fixations.

Yarbus [17] famously held that the scanpath could reveal what an observer was thinking about when viewing a scene, though that may not be as straightforward as he thought [18] (also see [19,20,21]). In the absence of eye tracking, people are surprisingly ineffective at knowing which part of an image they have looked at. We [22,23] investigated how well people could recall their own fixations after a brief period of scene search. In Võ et al [22]., observers were asked to perform a change detection task. They viewed a scene for 3 s and then saw a new version of the scene and were asked what had changed. On 25% of trials, after viewing the first scene for three seconds, observers were asked to click on 12 locations that they thought they had fixated. Humans make voluntary eye movements about 4 times per second; hence, 12 fixations in 3 s. People were not random, but the results show that observers’ memories for where they had looked in the scene were no better than their guesses about where someone else might have looked in the same scene. That is, you might know that it would be reasonable to look at the coffee mug on the desk while viewing an office scene, but you have no privileged access to whether you actually looked at the mug. Kok et al. [23] went on to demonstrate that online feedback has only a marginal effect on memory for the scanpath. They used a gaze-contingent window during the search to highlight where observers were looking and it was still difficult for those observers to maintain a representation of where they had looked once the search was done.

**Figure 1:**
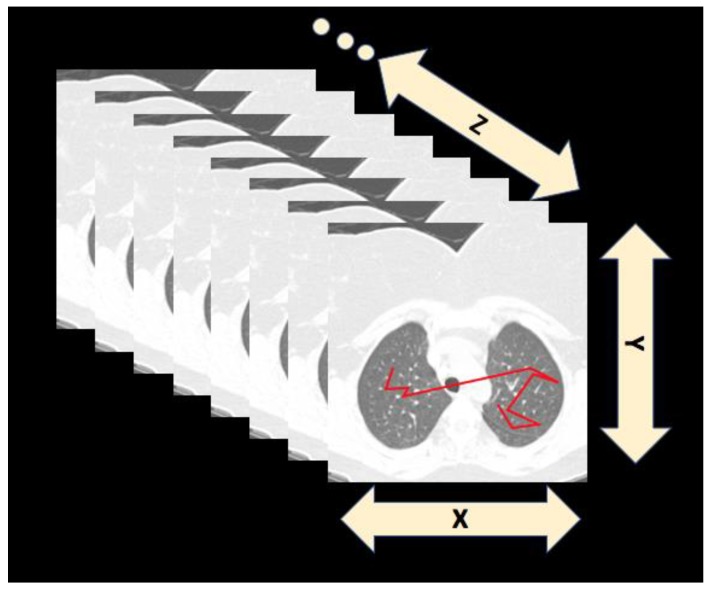
Eye tracking in 3D stacks of images means keeping track of the eye’s position in the XY plane and the depth (Z) of the currently displayed image.

Obviously, the failure to remember where you have looked before could contribute to errors in medical image reading because radiologists may also have poor representations of which areas they examined in an image. This could be particularly true for 3D volumes of image data like CT and MR. What have you really “looked at”, once you have scrolled through a sequence of images covering the volume of the chest, for example (see Figure 1)? This is closely related to the question of the UFOV, which is discussed below. With eye tracking, it would be possible to give feedback to a radiologist who is completing a search: For example, an eye-tracking computer might tell the observer, “You may have a good reason, but do you know you have not looked at *this* entire region?” or “You spent a lot of time looking at this spot. You did not label it as abnormal but it clearly grabbed your attention. Do you want to reconsider before you move on?” Kundel, Nodine, & Krupinski [24] found that giving feedback of this second sort to radiologists had a positive impact, and explorations of this type of intervention continue [25]. However, in other contexts, being told where you have fixated may not be that useful [26,27].

The increasing use of techniques like computed tomography (CT) has converted the measuring of the scanpath from a 2D to a 3D problem. Many modern imaging technologies create 3D volumes of image data. These are often rendered into ‘stacks’ of 2D images. The reader typically scrolls back and forth through the stack while examining the currently visible 2D image (see Figure 1).

Thus, the eyes move in the XY plane while movement in Z, depth in the stack, is typically controlled by the observer through the workstation.

**Figure 2:**
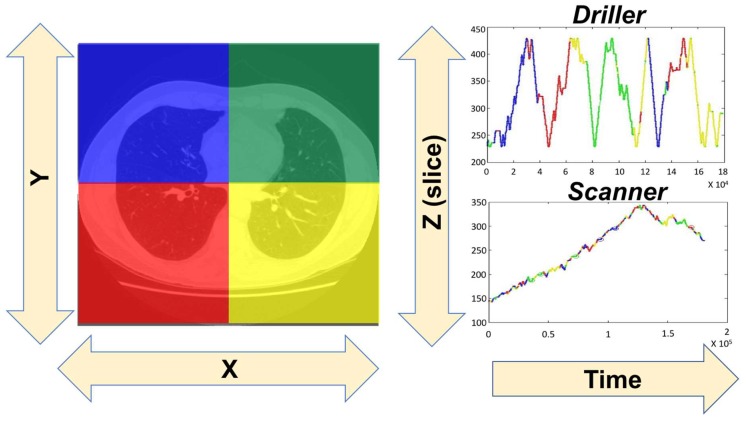
To visualize an approximation of the 3D scanpath through the lungs, position in the XY plane is coarsely color-coded into four quadrants (left-hand image). Depth (the slice in the stack) is plotted as a function of time-on-task on the right, with colors indicating the XY position. The terms “driller” and “scanner” are explained in the main text.

There has been a limited amount of research into search through 3D volumes of image data [28,29,30,31,32,33]. These 3D volumes of data represent an increase in the information/images that observers need to process. They also, necessarily, change observers’ eye movements patterns from a 2D search strategy in X&Y dimensions to a 3D search in X, Y, & Z. Drew et al. [34] had 24 radiologists search for lung nodules in stacks of images drawn from patients undergoing testing for lung cancer. As shown in Figure 2, to visualize the data, they color-coded the slices so that each quadrant had its own hue. Then they plotted that hue (a coarse measure of XY position) as a function of time in the search and the Z dimension, the slice in depth. They reported that radiologists could be coarsely split into two groups: “drillers” who moved rapidly in depth while staying in a relatively constant spot in the XY plane. In contrast, scanners moved slowly in depth while looking much more widely in the current XY plane.

We know that viewing lung CT images as an observer-controlled ‘movie’ is superior to viewing slices in a 2D array [35] but we don’t yet have enough data to know if we should be recommending ‘driller’ or ‘scanner’ strategies. Nevertheless, this type of scanpath research is an example of how eye movement recording can change our understanding of how the behavior of radiologists (or other experts) responds to changes in technology.

Once revealed, drilling and scanning seem like rather natural categories of scanpaths for 3D volumes of images. However, different technologies and different tasks may produce different oculomotor approaches. In breast imaging, the analog to the set of slices produced by CT is produced by digital breast tomosynthesis (DBT) [36], a somewhat different x-ray technology that also produces a stack of slices. At least one manufacturer explicitly trains radiologists to “drill” (albeit, not using that term). Nevertheless, when Aizenman et al. [28] measured scanpaths of radiologists reading DBT stacks, they found that the XYZ paths did not conform to the driller or scanner pattern from lung CT. Readers did tend to move rapidly back and forth through the depth of the breast, consistent with drilling, but they did not restrict themselves to one part of the breast in any rigorous manner. They seemed to scan and drill at the same time (“scrilling”?).

In thinking about possible differences between scanning in lung CT and breast tomosynthesis, it is worth noting that the different screening modalities are often used for different diagnostic purposes. For example, DBT is often used as a secondary diagnostic aid in addition to traditional mammography. But other 3D volumes of images, such as lung CT, serve as the primary screening tool. These different diagnostic tasks probably lead to very different scanpaths. Thus, it is important to search for general rules and also to check for those rules in multiple specific cases.

Returning to lung CT, if one looks more closely at either drilling or scanning behavior, one can see that readers are toggling quickly, back and forth between images. They may be looking for any items that might be nodules to see if they pop in and out of visibility as the observer toggles between a relatively few slices in the 3D stack. Other features, like vessels, snake through the image over many slices and would move rather than vanishing as the viewer moves a short distance through the stack. This shows one benefit of “toggling” between two (or more) nearly identical images, looking for change. Looking for change in this way is most effective when the toggled images are largely identical. Interestingly, there may be benefits even when images are not identical, as would be the case with a pair of mammograms of the same breast, taken at two different times. Drew et al. [37] asked radiologists to compare positive cases of mammograms with the negative prior exams acquired 2-3 years earlier from the same patients. Radiologists read the current and prior stimuli either in Side-By-Side mode or in Toggle mode. In Toggle mode, the radiologist could alternate between current and prior images at the same location on the screen. In Side-By-Side mode, current and prior images were visible at the same time on the screen. Drew et al. found that toggling produced a substantial improvement in time (~15%) and a small improvement in accuracy (~6%). The time benefit seems to result from the reduction in the number of required eye movements. In side-by-side viewing, readers made many saccades between the two images. Even though saccades are “cheap”, enough of them can add up to a real cost in time. The possible benefits of toggling for accuracy deserve further study.

## 3. The Useful Field of View (UFOV)

**Figure 3:**
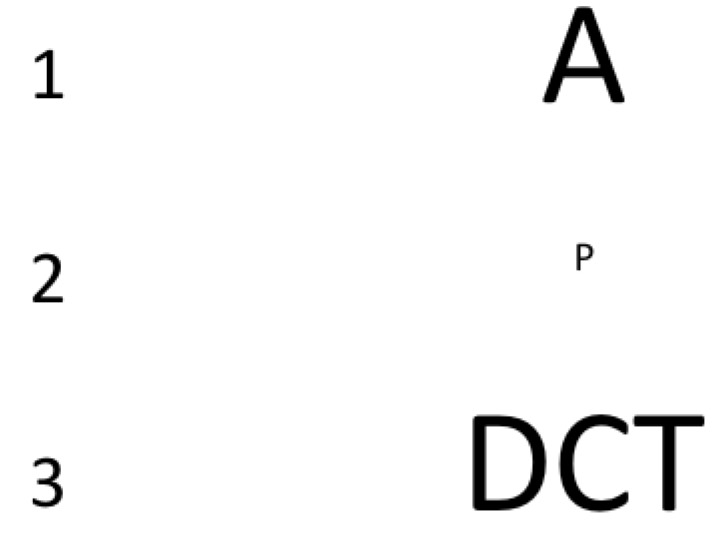
Acuity and crowding limit the UFOV.

Scanpaths raise an interesting problem. It is self-evident that observers move their eyes around an extended scene in order to see it better. But what exactly does that mean? It is clear that, as you fixate on this word *here, right now*, you can see the whole screen or page in front of you, but if your task is to read letters, your useful field of view (UFOV) is much smaller. You can only actually do that task in a narrow area around the current point of fixation. Some constraints on the UFOV are based on fundamental visual properties. Turning to Figure 3, if you fixate on the “1”, you can read the letter “A” but if you fixate on “2”, the decline in acuity away from the point of fixation will probably mean that you cannot read the letter “P”. If you fixate on 3, you will find that it is hard to resolve the letter “C” in the middle of the string “DCT”, even though it is the same size as the clearly visible “A” in Line 1 above. In Line 3, the problem is crowding [38,39], a reduction in our ability to process/recognize one object if it is surrounded by others.

**Figure 4:**
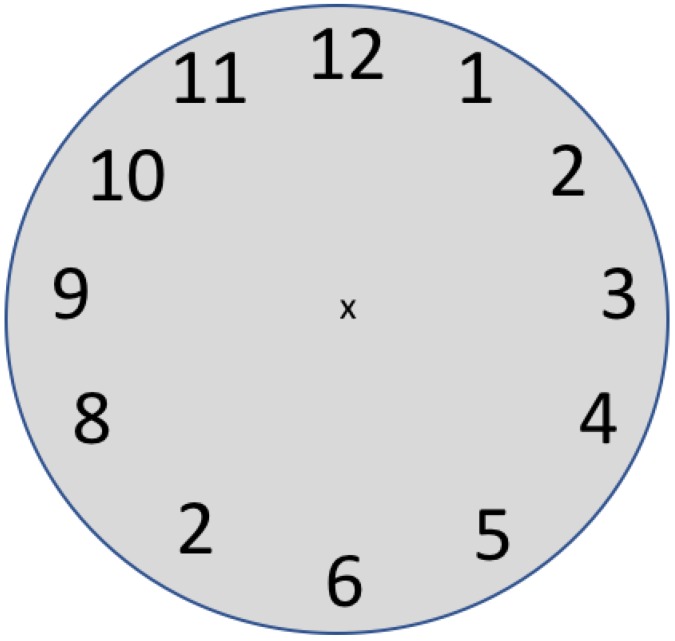
Fixate on the ‘x’ at the center and find the incorrect number.

Beyond these basic visual limits, there is also an important attentional limit on the UFOV (e.g., [40]). This is illustrated in Figure 4.

If you fixate on the ‘x’ at the center of this clock face, you should note that you can read each number in turn. If, however, you need to determine which number is out of sequence, you may find that a single fixation of a quarter to a third of a second is not adequate to do the task. If you were lucky, the ‘2′ in the 7 o’clock position was inside your UFOV for this task at this moment. If not, if you only had one fixation, you would have missed it. 

The same constraints that shape the UFOV in tasks like those illustrated in Figure 3 and Figure 4, will also apply in medical image search. When radiologists search a breast or a lung for a possibly cancerous mass, they will have a UFOV specific to the current image and task (e.g., the UFOV will be larger if the current target is larger). That clinical UFOV, like any other, will be affected by acuity, crowding and attentional factors. Thus, having an estimate of the UFOV can help us to understand whether the radiologist did or did not “look” at the whole image. 

Sanders [41] was an early pioneer in this area, investigating what we are calling the UFOV though he preferred the term “functional visual field” (FVF) [42,43]. Sanders divided the visual field into three attentional areas: the stationary field, in which people can process information without moving their eyes; the eye field, in which eye movements but not head movements are required to sample the information; and the head field, in which head movements would be necessary. He found that, in a target detection task, observers barely made any eye movements when the target was presented within 30 degrees. What we have learned, over the subsequent 50 years of attention research, is that measuring the UFOV for almost any task will involve more factors than those most extensively discussed by Sanders [41]. The size of UFOV will interact with the type of images, the properties of the target and its surroundings, and with human visual and attentional capacities. In a study of searching for low contrast lung nodules, Kundel and colleagues [44] reported that “The visual field size that is most effective in detecting nodules during search has a radius of 3.5 degrees visual angle. Nodule detection may be limited by basic neurologic constraints on human scanning performance”. In a separate study, Carmody et al. [45] asked observers to look for a nodule in chest x-ray films. The images were presented only for 300 msec to simulate a single fixation. They found that detection rates dropped by one-half when the nodules were presented at 5 degrees from the fixation. Apparently, it is hard to establish a reliable UFOV measure, even if the task is restricted to something as straight-forward as search for nodules in chest x-ray. Thus, any estimate, based on a scanpath, of how much of an image was examined should be treated with caution. It will be based on assumptions about the size of UFOV. That said, statements about *relative* coverage are more convincing. Unless there is some reason that the UFOV might change between conditions, it is reasonable to use scanpath data to say that observers looked at more of a scene under condition A than under condition B.

UFOV questions certainly do not become simpler when the stimuli are 3D volumetric images, such as lung CT. In one of very few studies, Ebner et al. [46] have reported that the nodule detection rate on chest CT was highly correlated not only with the size of UFOV, as measured by the nodule eccentricity (“transverse distance” in their paper), but with the nodule size and local lung complexity. There are essentially no data on the UFOV in DBT to say nothing of other tasks such as CT angiography where complex 3D image data changes over time. If it was important to know what percentage of an image set was examined in some specific task, UFOV measures would need to be made for that task. It is probably more useful to ask slightly different questions, based on the scanpath. For example, when should we be concerned that a reader is not looking at enough of an image?

Thus, with the cautions described above, it is possible, given some UFOV assumptions, to use scanpaths to make statements about how much of an image or a case has been examined. However, it is by no means clear how much coverage is enough. The unthinking answer is that we would hope that the radiologist would look at the “whole image” but that is clearly incorrect. Suppose you walk into the kitchen to look for a pepper grinder that is, in fact, not present. How much of the kitchen should you look at before declaring the object to be absent (akin to declaring the breast to be normal)? Looking at “everything” would be foolish. You, as a kitchen expert, know that the pepper grinder is never on the floor, even if it could be. On the very rare occasion that it is on the floor, you might fail to find it but, most of the time, not looking at “everything” is sensible. Similarly, an expert radiologist will know that there are some parts of an image that do not require fixation. Indeed, one of the oculomotor hallmarks of developing expertise is a tendency to look at less of the image [47,48]. We can see the utility of learning what not to look at in a study by Rubin et al. [49] where they found the radiologists on average search only 26% of the lung parenchyma yet encompass 75% of nodules in their search volume”. This applies beyond radiology; for example, in Dermatology [50]. Other oculomotor metrics also change as expertise develops [51] but the pruning back of the scanpath is an important and general sign of expertise. 

Returning to the idea of using eye movement feedback, discussed above, if the scanpath is to be used to warn the expert that some areas of the image are unexamined, those warnings should acknowledge that some areas simply do not need to be examined. Thus, it would be foolish to build a system that would insist that a reader fixate every part of an image before allowing the reader to move on to the next case and it might be clever if an artificial intelligence (AI) system could learn where in the image/scene a target might be and where it could never be.

## 4. Scanpath Signatures of Errors in Medical Image Perception

**Figure 5:**
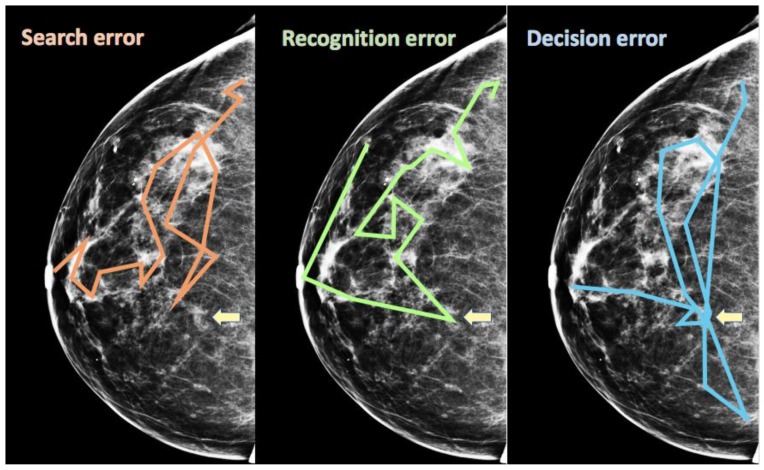
Three types of false negative (miss) errors, as proposed by Kundel and colleagues.

The motivation for eye movement feedback of the sort described above would be to prevent false negative (miss) errors in search. That implies that readers miss findings when they do not look at them. Not looking at a target is certainly one reason that the target might be missed, but it is not the only reason. In medical image perception, Kundel and colleagues developed a useful taxonomy of false negative errors, based on eye movement recording. For example, Kundel, Nodine & Carmody [52] recorded and analyzed eye movements from clinicians who were searching chest x-rays for lung nodules. They argued that clinicians’ eye movements could be used to distinguish three types of errors: Search, recognition, and decision errors. In various works, the terminology has varied somewhat but the core idea has remained about the same. In Figure 5, we will use those terms to describe the taxonomy. The scanpaths in Figure 5 are invented for purposes of illustration. Suppose that the yellow arrow is pointing to a finding in the breast that should be reported as suspicious. Kundel and colleagues argued that there were three different ways that this target might be missed. In a search error, the target is never fixated. A recognition error is said to occur when the eyes fixate on the target briefly and then move on, with no indication that the reader noted anything of interest. Finally, multiple and/or long fixations on a target indicate a decision error, if the reader still fails to report the finding. This pattern indicates that, implicitly or explicitly, the reader knew that this spot deserved attention but then the reader made the wrong decision and did not mark the spot as abnormal. In their lung nodule study, Kundel, Nodine & Carmody found that clinicians made about 30% search errors, 25% recognition and 45% decision errors. Similar proportions are found in a variety of studies (e.g., [47,53,54]).

How does this taxonomy of errors apply to 3D volumes of image data? The short answer is that the correct studies have not been done, but there are some hints. A major reason for moving to a 3D modality (e.g., lung CT) over a 2D one (chest x-ray) is that findings that are ambiguous in 2D are clarified by 3D. A downside of the move is that the increase in images leads to an increase in the time per case and to pressure to move quickly through the images. One might suspect that these factors would decrease the proportion of decision errors while increasing the proportion of search errors in which the target was never fixated. Drew et al. [34] found the signs of such a shift and a report by [49] that readers only search 26% of the lung in lung CT also points in that direction. The topic deserves further study. A more complete review of the work on specific types of search errors can be found in [12].

## 5. Incidental Findings and Satisfaction of Search Errors

Two, possibly related varieties of errors deserve some further mention. “Incidental findings” are findings that may be clinically significant but are not the primary target of the clinician’s search. A lung nodule would be a primary target in a search for signs of lung cancer. The same lung nodule would be an incidental finding if it was noticed in the course of an exam to determine if the patient had pneumonia [55]. Radiologists are typically expected to report incidental findings [56]. There is considerable debate about reporting and management of incidental findings because many of them turns out not to require any action. Raising them to attention can cause unnecessary worry and unnecessary medical care [57,58,59,60]. On the other hand, not reporting a finding that turns out to be clinically significant is a potent source of malpractice suits [61]. It is important to note that radiologists are trained to detect incidental findings. They would not typically stumble on a finding by chance. They know what they are looking for and, indeed, may have specific search strategies designed to detect problems that are not the specific focus of the case.

Medical image perception researchers do not need to solve the issue around the management of incidental findings. We can focus on reducing the number of targets that are not found at all. Clinicians cannot successfully manage what they don’t see. What kind of errors are missed incidental findings? In the eye tracking study that produced the driller/scanner data as shown in Figure 2, an image of a gorilla was inserted into the final case. It spanned five slices in the stack of CT images and was easily detectable in a variety of control conditions. Nevertheless, 20 of 24 radiologists failed to notice it [62]. The gorilla was chosen as a stimulus because it is the iconic stimulus [63] for studying the phenomenon of “inattentional blindness” [64]. The experiment showed that expertise does not immunize the expert against inattentional blindness, even when the stimuli are the subject of the expertise. When radiologists are looking for a small, round, white nodule, they are likely to miss a big visible gorilla right in front of their eyes because attention will be guided to the wrong set of basic color and shape features for gorilla detection [65].

Since the observers were being eye tracked, it was possible to apply the Kundel taxonomy to these miss errors (understanding that there are only 20 data points in total here, from the 20 miss trials). Observers spent nearly 6 s on average looking at the five slices that contained the gorilla. On average, they fixated on the gorilla itself for an average of 329 msec. Thus, most of these do not appear to be search errors. These appear to be recognition errors where the eyes landed on the gorilla but the strange identity of the item did not register with the observer. It seems quite unlikely that these could be decision errors. Of course, this does not mean that *all* misses or even most incidental finding errors are recognition errors. However, the result does make the point that an expert can look at something very odd in an image and, if looking for something else, fail to note the oddity.

As multiple radiologists have pointed out, gorillas are not an ideal model for incidental findings. When a radiologist misses a nodule while looking for pneumonia, she knows that lung cancer is something that can plausibly happen in a lung. Gorillas do not happen in lungs. In more recent work, we have developed a lab analog of incidental findings in which non-expert observers reliably miss over 30% of targets that they know that they are looking for [66]. Observers are looking for three specific images and, at the same time, for any members of any of three broad categories, like animals, hats, or fruit. They find the specific targets with ease but make large numbers of errors on the categorical targets. We don’t yet know if observers fixate the targets that they fail to detect because the eye tracking experiments have not been done. The results of the gorilla study would imply that we will find that observers can fixate on an elephant and still fail to report having found an animal.

Satisfaction of Search (SoS) errors are a class of false negative errors that are somewhat similar to incidental finding errors in the sense that, in both cases, one target, either the one you are searching for or the one has been found, interferes with the detection of another. In the SoS case, finding one target in an image makes it less likely that observers will report a second target compared to cases where only the second target is present [67]. The name comes from the original account of the source of these errors. It was initially proposed that, having found one target, observers were “satisfied” and, thus, abandoned the search too soon, before finding the second target. Subsequent research suggests that this theory is not correct [68,69,70] but the name has persisted, though Cain et al. [71] have tried to get the field to shift to “subsequent search misses (SSM)”.

There are two groups who have conducted the most extensive work on SoS: Berbaum and his colleagues (reviewed in [72,73]) and Cain, Adamo, Mitroff and colleagues [71]. Here, we want to focus on what eye movements can tell us about SoS errors. Kundel, Nodine, and their co-workers concluded that most SoS errors were recognition errors. The observers fixated on the missed items but only relatively briefly. If they spent a longer time, they tended to find the target [70]. Berbaum et al. [74] found that the type of task made a difference in x-ray studies of the abdomen. For some classes of radiologic exam, they found that the SoS errors tended to be search errors where the reader did not look in the right place. For other tasks, like Samuel et al. [70], they found a large proportion of recognition errors. On the other hand, in an eye tracking study of chest x-rays Berbaum et al. [75] found very few search errors, 35% recognition errors, and 58% decision errors. In that study, the readers apparently looked at the targets for some time and decided not to call them targets. Using a very different task, search for low contrast T’s among L’s, with a non-expert population of observers, Cain et al. [71] found search errors to be the largest category (37.8%), with the second largest category, at 24.3%, being a new type of error that they called “resource depletion errors”. They define resource depletion errors as errors that arise when the first target depletes working memory resources that could be used to find the second target. Recognition and Decision errors together account for only 20% of errors in their study. Clearly, SoS (or SSM) errors are not the product of a unitary mechanism in search. As shown in [71], there is a taxonomy of these errors, as there is a taxonomy of errors in search more generally.

## 6. Scene Gist and Medical Scene Gist

There are other emerging uses of eye tracking in medical image perception. For example, Drew and colleagues have been using eye tracking to address the effects of interruption on radiologists [76,77]. However, we want to finish this brief review with some consideration of medical image searches that involve little or no oculomotor activity. To quote Kundel [78], “Clearly much of what happens in perception precedes exhaustive visual scanning of the image.” An important part of the Kundel-Nodine model of search in medical images is a “holistic” stage lasting about a second [79,80,81] during which the observer might be processing the whole image without needing to move the eyes. Much of the basis for proposing this holistic stage comes from eye tracking studies that often show the eyes of experts moving to the target almost immediately [82,83].

Basic research in visual attention would divide these holistic effects into more than one component. “Covert attention” can be shifted more rapidly and more frequently than the eyes [84,85,86] so attention might have reached a target before the eyes had a chance to move. The first eye movement might be simply confirming what had been found. Second, a set of basic features guides the deployment of attention and the eyes [87,88]. Thus, in a search for a lung nodule, attention will be guided to a location that contained the small, white, and round features which are characteristic of a nodule. Again, the first eye movement might go to a target because the target’s features provided successful guidance about where to best deploy the eyes.

Kundel and Nodine [79] investigated whether there is any useful information available before the eyes move by asking radiologists to evaluate X-rays with only a 200 msec exposure to the images. With these particular stimuli, expert performance was nearly perfect with unlimited viewing. Surprisingly, with just a 200 msec glimpse of the image, their classification accuracy was still about 70%. In their following study, Carmody, Nodine and Kundel [89] systematically varied the exposure duration to test how detection performance changes over the first half-second of exposure. They found that, across the different level of image visibilities, the performance reached an asymptote after 240 msec. Moreover, even for the least visible cases, there was still substantial information available in the first quarter of the second. This global analysis not only occurs in lung screening, but also in the screening of mammography [90]. The Kundel-Nodine group argues that a major part of the development of expertise is a growing ability to do this holistic processing. They invoke this holistic stage to explain why experts make fewer eye movements.

Interestingly, there is an aspect of this holistic stage of processing that does not serve to direct the eyes to the target. Evans et al. [91] showed radiologists a brief flash of a mammogram for durations of from 250 msec to 2 s and asked them to classify this case as normal or abnormal (Would you call back this patient?). They found that radiologists could perform at above chance levels at all stimulus durations, even those that did not permit a voluntary eye movement. Importantly, this awareness of abnormality did not appear to be based on visible features of a lesion because when radiologists were also asked to place a localizing mark on the most suspicious location on a white outline mask of the breast after the brief presentation of stimulus, their performance was not better than chance. This was true regardless of their rated degree of confidence that the presented image was abnormal. Readers were not simply getting lucky and fixating a lesion on a subset of trials. In a subsequent study, Evans et al. [92] showed that this global “gist signal” was not based on a break in the normal asymmetry between breasts nor was it a proxy for breast density, a known risk factor for breast cancer. In fact, radiologists were able to discriminate between normal and abnormal at above chance levels when the “abnormal” images were the images from the breast contralateral to the breast with overt signs of cancer. Since there is no lesion presented in the image, the observed performance cannot be due to a lucky fixation on a mass. Something about the texture of the breast tissue is abnormal and experts have become sensitive to that signal. Brennan et al. [93] repeated the experiment but with the “priors”, the mammograms acquired three years before the women developed overt signs of cancer. Even though there were no localized lesions in these priors, radiologists could still detect this gist signal at an above chance level. A signal that is available years before the cancer develops could be a useful imaging biomarker of cancer risk.

## 7. Conclusions

In this brief review, we have tried to show the usefulness of eye tracking for understanding how experts perform tasks like those involved in clinical radiology. A similar story could be told about many other expert domains. Scanpaths tell a story. The story may not be as clear as optimistic interpretations of Yarbus’ work might suggest, but the sequence of eye movements and the placement of fixations relative to targets tell us a lot about the processes of expert visual search. Eye movement recordings are of particular interest in analyzing errors and, we may hope, in testing efforts to reduce those errors. It is notable how many of the basic issues in this field were outlined and studied by Kundel, Nodine, and their group (as well as by other labs and earlier researchers). However, their path breaking work is not the end of the story. As long as the advances in medical imaging create new stimuli to improve medical interpretation, there are always new scientific questions that need to be investigated.
